# Efficient Vaccination

**Published:** 1897-12

**Authors:** Alex. W. Sterling

**Affiliations:** Atlanta


### CORRESPONDENCE.

### EFFICIENT VACCINATION.

ATLANTA, November 28, 1897.

DEAR SIR:—In discussing vaccination here I have been struck with certain differences in the procedure as compared with those of Great Britain. I do not refer to the fact that in the latter country vaccination is compulsory for every child under six months of age, but rather to the favorite methods of storing the lymph, and to the number of marks considered necessary to afford protection from smallpox. Here "points" appear to be the favorite method of storage, while in Britain these are considered liable to contamination, and it is held to be preferable to keep the lymph mixed with glycerine in Husband's glass tubes, each tube containing sufficient virus for one patient only. Care is also taken that the patient's arm is specially prepared, as if for any other operation. The Local Government Board contains among its regulations one to the effect that for all cases of primary vaccination such insertions of lymph should be made as will produce at least four separate good-sized vesicles, or groups of vesicles, not less than half an inch from one another. It has been abundantly proven in England that protection varies directly with the number of good marks up to a certain figure. As an example of the statistics which might be quoted to prove this, I might mention the following: From some 11,000 cases of smallpox having one vaccine cicatrix, 13.8 per cent. proved fatal; having two cicatrices, 7.7 per cent.; having three cicatrices, 3.0 per cent., and having four cicatrices, 0.9 per cent. Other observations dealing with large numbers of cases confirm the above. I notice that vaccination is about to be made for the present compulsory in Atlanta, and as from the hearsay evidence, which is all that I can claim, one mark is usually considered sufficient here, I thought that perhaps those who may not be already acquainted with them might be interested in these regulations of the English Local Government Board.

Yours truly, ALEX. W. STERLING, M.D.

